# Using classification and regression trees (CART) to investigate unknown ethnicity records in NHS emergency care data

**DOI:** 10.1136/bmjopen-2026-118200

**Published:** 2026-08-17

**Authors:** Grace Dean, Roy A Ruddle

**Affiliations:** 1 NHS England, Leeds, UK; 2 School of Computing and Leeds Institute for Data Analytics, University of Leeds, Leeds, UK

**Keywords:** Emergency Service, Hospital, Health policy, Electronic Health Records

## Abstract

**Abstract:**

**Objective:**

High-quality ethnicity data are necessary for tackling health inequalities. This study aims to support the improvement of ethnicity recording in emergency care data by (a) investigating variation in unknown ethnicity records, (b) exploring patterns of missingness between ethnicity and other variables and (c) identifying the variables most important in the recording of an unknown ethnicity.

**Design:**

Secondary analysis of the Emergency Care Data Set (ECDS).

**Setting and participants:**

National Health Service (NHS) and independent sector organisations who provide emergency care services in England. Includes all anonymised ECDS records from the financial year 2023/2024, excluding those with a ‘Null’ anonymised pseudo-NHS number or those patients announced as dead on arrival (24 167 154 records after exclusions).

**Results:**

Differences in the percentage of unknown ethnicity records were seen across ages, organisation types and attendance characteristics. An increase in the percentage of missing values was observed for 62 variables when ethnicity was unknown, with the largest increases shown by variables related to investigations and treatments received by the patient (15.31% and 14.74%). Provider site code was identified as the most important variable in recording ethnicity as unknown. Further analysis highlighted a subset of acute trusts and independent sector organisations that disproportionately contributed to unknown ethnicity records.

**Conclusions:**

This study has improved understanding of unknown ethnicity recording by exploring variation across demographics and organisation types and demonstrating an increase in missingness across other ECDS variables when ethnicity is unknown. Unknown ethnicity recording appears to be a site-specific problem, which should be addressed through improvements to the data collection processes of individual providers. These insights should be used to improve the quality of ethnicity coding within emergency care data, necessary for tackling health inequalities.

**STRENGTHS AND LIMITATIONS OF THIS STUDY**

- This study compared unknown ethnicity records in Emergency Care Data Set across demographics, attendance characteristics and organisation types.
- The ‘VizDataQuality’ Python package enabled the investigation of patterns of missingness between an unknown ethnicity and other variables.
- Classification and regression trees (CART) were used, via the ‘rpart’ R package, to identify the factors most important in recording ethnicity as unknown.
- Restrictions in computational power limited the data set to a random 50% sample when using the ‘VizDataQuality’ Python package, with further sampling required for the CART analysis.
- The study focused solely on the completeness of ethnicity data and therefore did not consider the accuracy of the data.

## Introduction

 Disparities in health outcomes of the COVID-19 pandemic^1 2^ highlighted the inequalities experienced by different ethnic groups.^3^ High-quality ethnicity data must be complete, self-reported, consistent and granular^4^ if used to tackle health inequalities.^5^ However, this remains a global challenge.^6–8^ In England, differences in the experience, access and outcomes of healthcare exist across different ethnic groups.^9–11^ The National Health Service (NHS) has committed to improving the quality of ethnicity data to address these inequalities.^12^ However, data quality remains a significant concern,^13^ with issues of completeness, accuracy and consistency reported across several NHS datasets.^14^

The Emergency Care Data Set (ECDS) is an anonymised patient-level data set for urgent and emergency care services in England.^15^ Approximately 15% of ECDS records lack valid ethnicity information.^16^ Data completeness is impacted by the categories ‘Not Known’ and ‘Not Stated’, which account for most unknown ethnicity records.^17^ While considered valid during data entry, these codes lack informative value and increase the proportion of missing data.^14^ Use of these codes reduces the usefulness of ethnicity data.^18^ This can negatively impact the accuracy of subsequent analysis, shown when modelling COVID-19 cases for different ethnicities.^19^

Unknown ethnicity codes can create representation bias within data. Representation bias occurs when the under-representation of specific group(s) of the population results in discriminatory outcomes^20 21^ and has been increasingly identified in healthcare studies.^22 23^ For example, a model developed to predict acute kidney injury using mostly male records (94%) had weaker performance for women compared with men.^24^ Missing data can create representation bias if skewed towards specific groups rather than being missing completely at random (MCAR).^25–27^ Patients from an ethnic minority background are more likely to have an unknown ethnicity recorded.^16^ Therefore, incorrect use of the ‘Not Stated’ and ‘Not Known’ categories may entrench existing health inequalities by preventing the data from accurately representing all groups of the population.

To improve ethnicity recording, it is crucial to understand the patterns and structure of the missing data. Missing data can hold useful information regarding existing biases within the data collection process.^28^ For example, completeness of an electronic health record is linked to the frequency of patient interaction with the health service, with sicker patients more likely to have a complete record.^25 29^

Furthermore, variation in unknown ethnicity records exists for patients admitted to hospital, with working-age patients and those with shorter hospital stays more likely to have an unknown ethnicity.^16^ Examining unknown ethnicity records can uncover valuable information about the factors that influence missingness.

Advanced data visualisation techniques can identify patterns and structure in the missing data. For example, interactive bar charts and heatmaps can highlight variables missing most often and those missing together.^28 30^ Moreover, missing data pattern classification can identify the patterns of missingness between variables, with purity calculations used to separate perfect and imperfect patterns.^31^

Machine learning methods can identify which variables contribute most to the missingness of other variables. Classification and regression tree (CART) models have previously been applied to an occupational health dataset with a large proportion of missing data (~63%) and used to analyse the structure of the missing data.^32^ The model identified the variable which best predicted the amount of missing data in each record and found the value of this variable which led to an increase in overall missingness.^32^ Moreover, CART models were used to analyse patterns of missingness in body mass index (BMI) data and identified subgroups of the dataset who were more likely to have a missing BMI than others.^33^ These techniques can be used to highlight variables associated with missing data and draw attention to subgroups who may be disproportionately impacted.

This study has three objectives: (a) investigate variation in unknown ethnicity recording, (b) explore the patterns of missingness between ethnicity and other variables and (c) identify which variables contribute to ethnicity being recorded as unknown. This work addresses an important research gap by evaluating the completeness of ethnicity data within recent emergency care records and providing a detailed assessment of variation in unknown ethnicity records. It applies advanced data visualisation and machine learning techniques to provide new insights that will enhance current understanding and aid the improvement of ethnicity recording within emergency care data.

## Methods

### Data

Introduced in 2017,^34^ the ECDS holds accident and emergency attendance data for urgent and emergency care services. Each record contains attendance details, including the activity type,^35^ reason for attendance and treatment received. Patient demographic information (eg, age, sex and location) is recorded alongside an anonymised pseudo-NHS number.^36^

All providers of urgent and emergency care are required to record a patient’s ethnicity based on their response to a series of monitoring questions.^37^ The codes match the 2001 Census definitions^38^ (online supplemental table 1). ‘Not Stated’ should be used when the patient has been asked the ethnic monitoring questions but chose not to declare their ethnicity.^16^ ‘Not Known’ should be used when the patient was not asked their ethnicity or was unable to answer.^16^ Any ethnicity records left blank by the provider during the data collection process appear as ‘Null’. For this analysis, ‘Not Known’, ‘Not Stated’ and ‘Null’ are categorised as an unknown ethnicity. All other codes are categorised as a known ethnicity.

National data for the 2023/2024 financial year were extracted and cleaned using Databricks (Runtime V.14.3 LTS) on NHS England’s secure data environment via a remote desktop service. Data cleaning involved removing records where the anonymised pseudo-NHS number was ‘Null’, because rows without an anonymised pseudo-NHS number cannot be validated, deduplicated or reliably linked. Rows were also removed where the patient was announced as dead on arrival.

Any columns relating to NHS England’s engineering process were removed as these were extraneous. The resulting dataset contained 24 167 154 records and 208 columns (online supplemental table 2). This included data for 189 organisations across six organisation types: acute trusts, ambulance trusts, community trusts, independent sector organisations, mental health and learning disability trusts and non-NHS organisations.

Analysis was conducted using either Databricks (Runtime V.14.3 LTS) or R-studio (R V.4.4.2) on NHS England’s secure data environment. Initial analysis highlighted data quality issues, with only two of the 54 mental health trusts, one non-NHS organisation and one of the 10 ambulance trusts recorded within ECDS. These organisations were excluded from further analysis. ‘Null’ values across all columns were retained to allow for the patterns of missingness between ethnicity and all other variables to be explored.

### Analysis

Analysis was split into three interlinked subsections. First, exploratory analysis examined the completeness of ethnicity and variation in unknown ethnicity records across different groups of the population (see ‘Results: Variation in unknown ethnicity recording’). Unknown ethnicity records were investigated by organisation type, the category of unknown ethnicity (‘Not Stated’, ‘Not Known’ or ‘Null’) and across different patient demographics and attendance characteristics.

Second, the structure of missing data for all ECDS variables other than ethnicity was analysed (see ‘Results: Patterns in missing data’). The number of missing values and the absolute difference in the percentage of missing values between records with known and unknown ethnicity was calculated for each variable. This showed the overall missingness for each variable and highlighted variables where missingness increased when ethnicity was unknown. The analysis on the structure of missing data was repeated for the different categories of unknown ethnicity (‘Not Stated’, ‘Not Known’ or ‘Null’) and organisation types.

The Python package ‘VizDataQuality’ was used to investigate the patterns of missingness between ethnicity and other ECDS variables.^31^ A pattern refers to the structure of the missing data and helps indicate if MCAR, linked to another value (Missing At Random, MAR) or due to something unobserved (Missing Not At Random, MNAR).^28^ Variables can be missing in specific patterns: (a) block pattern, where identical sections are missing together; (b) disjoint pattern, where variables are missing in different records and (c) monotone pattern, where all subsequent variables are missing if one variable is missing.^31^ Pearson correlation coefficients were used to calculate the purity of the pattern, with values equal to or greater than 0.95 considered significant.^31^ Patterns of missingness were calculated for the different categories of unknown ethnicity (‘Not Stated’, ‘Not Known’ or ‘Null’) and organisation type.

Third, a CART model was applied using the ‘rpart’ R package to identify the variables most important in recording ethnicity as unknown^39^ (see ‘Results: Investigating variable importance’). The model constructs a decision tree to explain a target variable from many explanatory variables.^40^ CART models are suitable for analysing the ECDS for several reasons. First, they are non-parametric, so can handle large datasets containing multiple categorical variables.^41^ Second, the package offers built-in cross-validation and pruning, which mitigates the challenge of overfitting.^42^ Thirdly, CART uses the ‘surrogate’ method to handle missing data; if a chosen variable contains missing values, then the model selects a similar variable for the split.^42^ This allows all records to be included in the analysis, irrespective of missing values. This is useful when investigating the structure of missingness^32^ and can alleviate potential bias being introduced by complete case analysis.^33^

Based on insights from the exploratory analysis and input from domain experts, 20 variables were selected for the CART model (online supplemental table 3). The variables were chosen to ensure the model would remain interpretable to NHS England and provider staff, enabling them to take meaningful action based on the results. Variables, therefore, focused on patient demographics and attendance characteristics, such as age, sex, deprivation, chief complaint on arrival and time in department. Variables related to NHS operational, financial or data engineering procedures (eg, Commissioner_Reference_Number and SUS_HRG_Code and Service_Agreement_Provider) were excluded. Variables with similar definitions were also excluded; for example, Age_at_Activity_Date, Fractional_Age_at_Arrival and HES_Age_at_Arrival were comparable to Age_at_Arrival and therefore considered redundant and removed. Similarly, many variables related to the geographical location of the provider and the patient’s home address (eg, Der_Postcode_LSOA_2011_Code, Der_Postcode_MSOA_2011_Code and Der_Postcode_CCG_Code) were considered redundant and removed.

Separate datasets were created for acute trusts, community trusts and independent sector organisations to prevent the imbalance in number of records per organisation type from concealing insights relating to the smaller organisation types. Each dataset was shuffled and split into 70:15:15 training, validation and test. Random over-and-under-sampling was applied using the ‘ROSE’ R package^43^ to address the imbalance in the target variable. The primary evaluation metric was balanced accuracy, which enabled fair comparison of accuracy while accounting for the imbalance in the data. The sensitivity and area under the curve (AUC) were also calculated to demonstrate the model’s ability to correctly predict an unknown ethnicity and discriminate between classes.

### Patient and public involvement

Neither patients nor the public were involved in the design, or conduct, or reporting, or disseminationplans of this research. Transparent Reporting of a multivariable prediction model for Individual Prognosis Or Diagnosis (TRIPOD) reporting guidelines were followed where appropriate.^44^

## Results

The following results are structured around the three objectives: (a) to investigate variation in unknown ethnicity recording, (b) to explore patterns of missingness between ethnicity and other ECDS variables and (c) to identify which variables are the most important in ethnicity being recorded as unknown.

All records were used to explore variation in unknown ethnicity recording. Due to restrictions in computational power, a random 50% sample was used for initial exploration of the patterns of missingness (12 082 016 records and 208 variables). Specific organisation type datasets were created for the remaining analysis. The community trust and independent sector datasets contained 468 039 rows and 680 208 rows, respectively, comparable in proportion to the original dataset. The acute trust dataset required further sampling, equating to 10% of records for acute trusts from the original dataset (2 161 282 rows).

### Variation in unknown ethnicity recording

Exploratory analysis showed that 14.91% of ECDS records had an unknown ethnicity; 7.75% were coded as ‘Not Stated’, 5.67% as ‘Not Known’ and 1.50% as ‘Null’ (online supplemental table 4). Differences were observed between organisation types. There were 126 acute trusts, which represented the majority of ECDS records (89.39%) (table 1). There were fewer independent sector organisations (45) and community trusts (14), representing 5.62% and 3.88% of ECDS records, respectively. Independent sector organisations had the highest percentage of unknown ethnicity records (39.72%), followed by community trusts (19.62%), while acute trusts had the lowest (12.58%).

**Table 1:** The total number of ECDS records and the percentage of ECDS records with an unknown ethnicity by organisation type

Organisation Type	ECDS Records		ECDS Records with an Unknown Ethnicity		
	Percentage	Count (n)	Unknown type	Percentage	Count (n)
**Acute Trusts**	89.39%	21 602 076	Not known (%)	4.01%	865 288
			Not stated (%)	7.58%	1 637 269
			Null (%)	0.99%	214 267
			**Total (%**)	**12.58%**	**2 716 824**
**Ambulance Trusts**	0.06%	13 982	Not known (%)	0.34%	47
			Not stated (%)	1.77%	247
			Null (%)	0%	0
			**Total (%**)	**2.10%**	**294**
**Community Trusts**	3.88%	936 582	Not known (%)	16.54%	154 883
			Not stated (%)	3.07%	28 719
			Null (%)	0.02%	166
			**Total (%**)	**19.62%**	**183 768**
**Independent Sector Organisations**	5.62%	1 359 337	Not known (%)	25.14%	341 675
			Not stated (%)	13.73%	186 637
			Null (%)	0.86%	11 669
			**Total (%**)	**39.72%**	**539 981**
**Mental Health and Learning Disability Trusts**	1.05%	252 616	Not known (%)	3.07%	7760
			Not stated (%)	7.62%	19 250
			Null (%)	53.55%	135 276
			**Total (%**)	**64.24%**	**162 286**
**Non-NHS Organisations**	0.00%	30	Not known (%)	96.67%	29
			Not stated (%)	0%	0
			Null (%)	0%	0
			**Total (%**)	**96.67%**	**29**

ECDS, Emergency Care Data Set.

The category of unknown ethnicity varied by organisation type. ‘Not Stated’ was the most common category for acute trusts (7.58%), representing nearly twice as many records as the ‘Not Known’ category (4.01%) (table 1). In contrast, ‘Not Known’ was the most common category for independent sector and community trusts (25.14% and 16.54%). This suggests fewer patients were asked their ethnicity in a community or independent sector setting compared with an acute setting, while patients were more likely to choose not to declare their ethnicity within an acute setting than not be asked. ‘Null’ represented the smallest percentage for records for all three organisation types.

Variation in unknown ethnicity recording was also observed across different demographic and attendance characteristics. Younger patients had a higher percentage of unknown ethnicity records compared with older patients; those aged 21–30 had 17.74% unknown ethnicity records, which gradually declined by age group to 10.05% for those aged 91–100. Differences were also observed based on the severity of the patient’s condition and frequency of attendance. Patients who presented with a minor condition^45^ had a higher percentage of unknown ethnicity records (19.09%) compared with those who required very urgent level emergency care (10.76%).^45^ Similarly, patients who spent less than 1 hour in the department had a higher percentage of unknown ethnicity records than those waiting between 24 and 48 hours (18.14% and 8.82%, respectively), and those who presented at a Minor Injury Unit or Walk-in Centre had a higher percentage than those who presented at an accident and emergency (A&E) facility (37.95% and 11.36%, respectively). Lastly, patients who attended an emergency department once within the 2023/2024 financial year had a higher percentage of unknown ethnicity records (17.17%) compared with patients with more than 10 attendances (9.35%). This shows that patients who presented with a minor condition, spent less time in the department or visited an emergency department less frequently were more likely to have an unknown ethnicity record than those who presented more often or required more care.

Overall, the exploratory analysis demonstrated variation in unknown ethnicity recording by age, organisation type and for different attendance characteristics. Acute trusts had the lowest percentage of unknown ethnicity records and used ‘Not Stated’ most often, while independent sector and community trusts had higher rates of unknown ethnicity records and used ‘Not Known’ more frequently. Younger patients and those presenting with less severe conditions were more likely to have an unknown ethnicity recorded compared with older patients or those who present with a more serious condition.

### Patterns in missing data

Next, the structure of all missing data was analysed. First, the number of missing values was calculated for each variable; 153 variables (73.56%) had missing values. Of these, 77 variables had missing values in less than 1% of records, and 18 variables had missing values in more than 99% of records. These 95 variables were removed, leaving 112 variables to be analysed alongside ethnicity.

**Figure 1:**
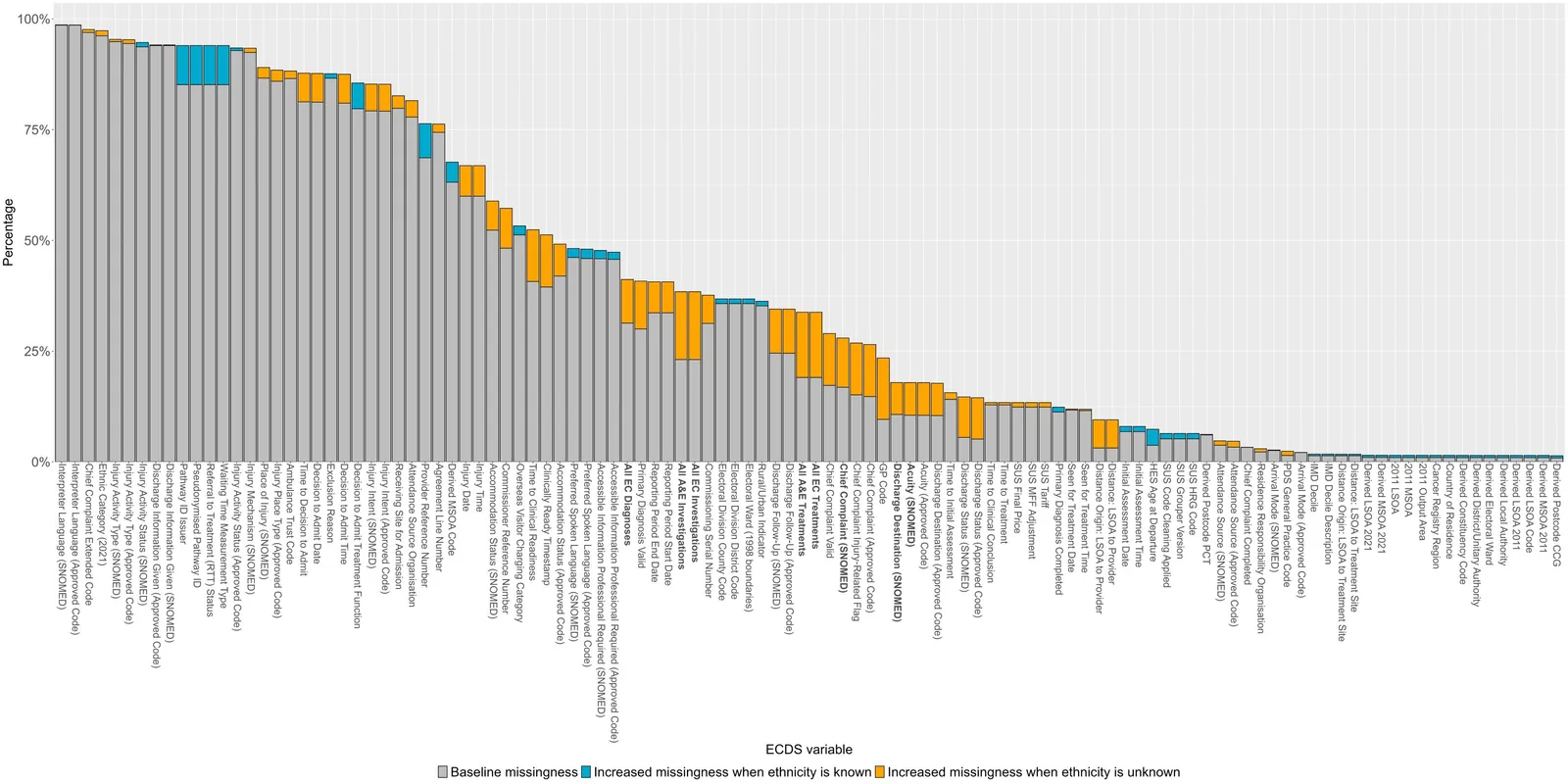
The percentage of missing values for each variable in ECDS. The orange and blue bars show the absolute difference in the percentage of missing values between records with known and unknown ethnicity. The orange bars represent the increase in the percentage of missing values when ethnicity was unknown, while the blue bars represent the increase when ethnicity was known. 62 variables saw an increase in the percentage of missing values when ethnicity was unknown (orange bars) and 49 variables when ethnicity was known (blue bars). The average increase in the percentage of missing values was approximately three times higher when ethnicity was unknown (5.85%) compared with known (1.79%). ECDS, Emergency Care Data Set.

The absolute difference in the percentage of missing values between records with known and unknown ethnicity was calculated for each variable (figure 1). 62 variables showed an increase in the percentage of missing values when ethnicity was unknown, more than the number of variables with increased missingness when ethnicity was known (49). This indicates that missing data across other ECDS variables are more prevalent when ethnicity is unknown. Moreover, the average increase in the percentage of missing values was approximately three times higher when ethnicity was unknown (5.85%) compared with known (1.79%). This shows that the extent to which data are missing across other ECDS variables is greater when ethnicity is unknown.

The largest increase in the percentage of missing values when ethnicity was unknown (15.31%) was observed in two variables, Der_AEA_Investigation_All and Der_EC_Investigation_All (figure 1). Both variables are related to the clinical investigations completed during a patient’s attendance^46 47^ and are missing together in a pure block pattern. The second largest increase (14.74%) was seen in Der_AEA_Treatment_All and Der_EC_Treatment_All; both are related to the treatment a patient receives^48 49^ and were missing in a pure block pattern. Other variables which saw an increase in missingness include the chief complaint the patient presented with (11.11%), the patient’s diagnosis (9.85%), the severity of the patient’s condition on arrival (7.37%) and the place of discharge (7.19%).

Larger increases in missingness for these variables were observed when ethnicity was ‘Not Known’ compared with ‘Not Stated’. For example, Der_EC_Investigation_All saw a 21.67% increase in the percentage of missing values when ethnicity was ‘Not Known’ compared with a 7.97% increase for ‘Not Stated’. This shows that the missing data for these variables are greater when the patient was not asked their ethnicity, compared with when they chose not to declare it.

**Figure 2:**
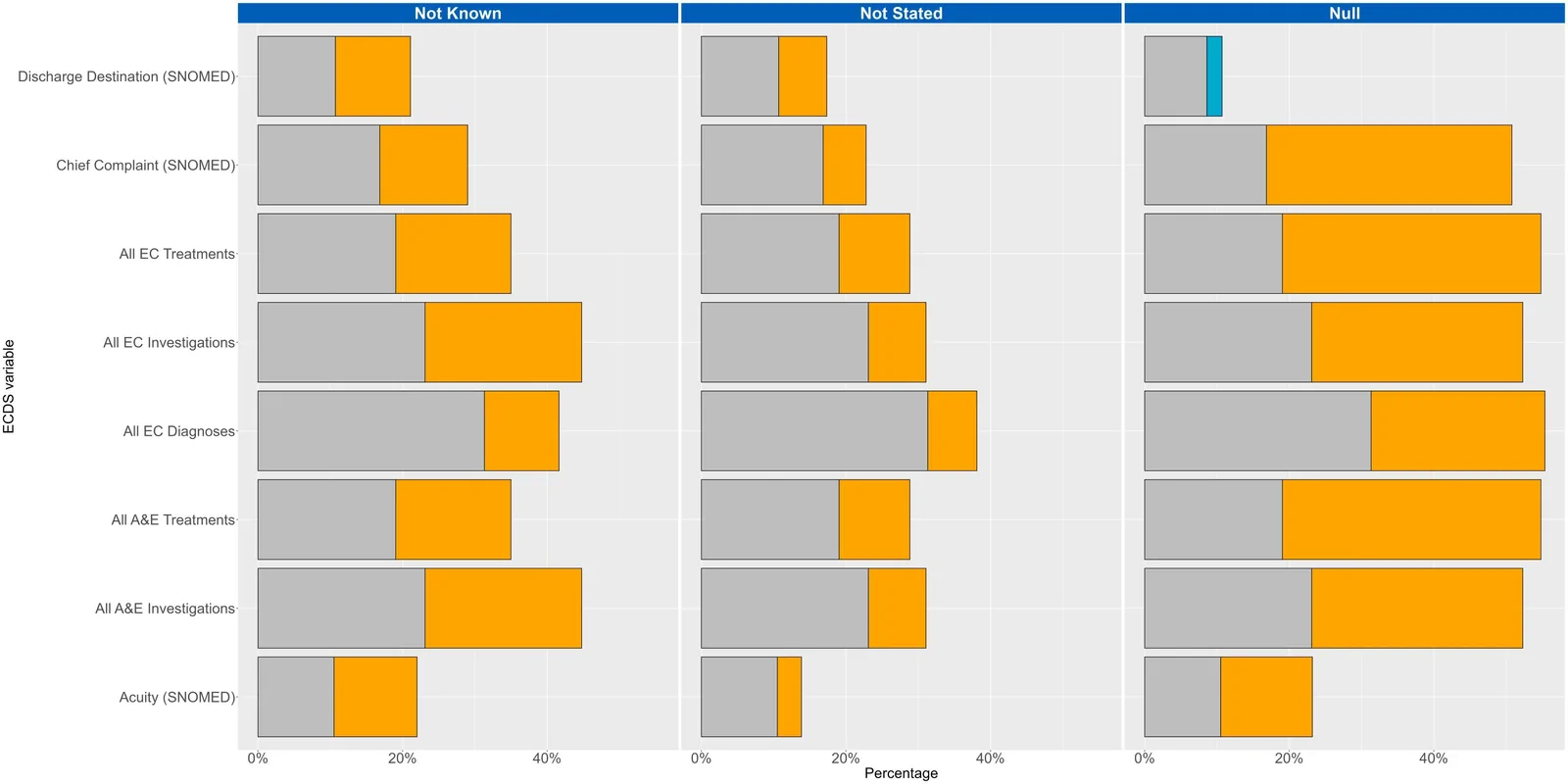
The percentage of missing values for eight variables within ECDS by organisation type. The orange and blue bars show the absolute difference in the percentage of missing values between records with known and an unknown ethnicity. The orange bars represent an increase in the percentage of missing values when ethnicity was unknown, while the blue bars represent an increase when ethnicity was known. A&E, Accident and Emergency; ECDS, Emergency Care Data Set; EC, Emergency Care.

Differences were also observed across the organisation types (figure 2). Independent sector organisations saw the largest increases in missingness for these variables, ranging between 5.71% and 21.11%. In contrast, community trusts saw the smallest increases, ranging between 0.55% and 3.62%. Moreover, four of the eight variables saw a decrease in the percentage of missing values when ethnicity was unknown for community trusts. This demonstrates variation between the organisation types regarding an increase in the missingness of other ECDS variables when ethnicity is unknown.

Overall, the pattern of missingness analysis has shown that missing data in other ECDS variables are more common and more extensive when ethnicity is unknown, compared with when it is known. The analysis has also revealed differences in the structure of missingness between the different categories of unknown ethnicity and between the different organisation types.

### Investigating variable importance

CART analysis was used to identify which variables were most important in recording ethnicity as unknown (online supplemental table 5). The relative importance for each variable demonstrates both its predictive power and contribution as a surrogate variable.^42^ Therefore, not all variables considered important are included in the decision tree, but they do provide insight into the structure of the data.^40^

The primary evaluation metric was balanced accuracy, which evaluates a model’s ability to correctly classify observations by averaging its sensitivity and specificity. This ensures that the model’s ability to identify both positive outcomes (sensitivity) and negative outcomes (specificity) are equally weighted when assessing performance, which is particularly valuable when dealing with class imbalance.

**Figure 3:**
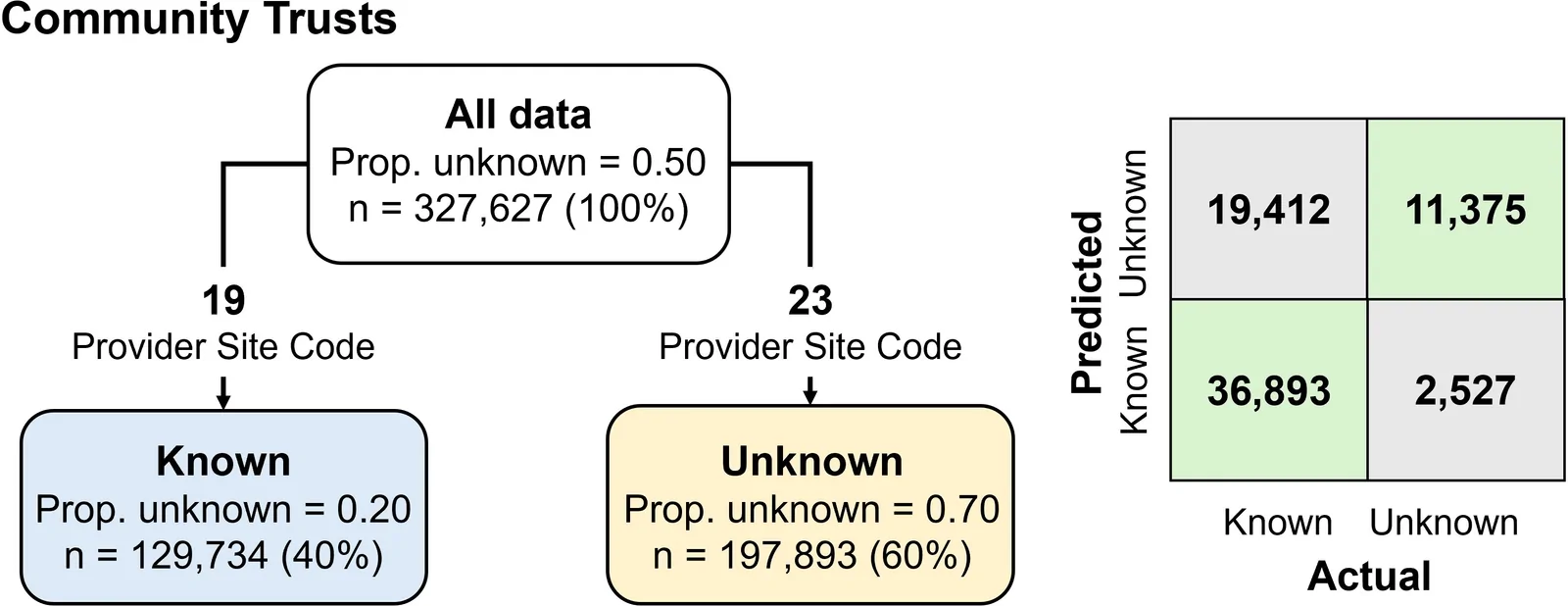
Classification and regression tree (CART) analysis for the community trusts, showing provider site code as the most important factor in recording ethnicity as unknown. Each node shows the proportion of unknown ethnicity records (prop. unknown) and the total number of records (**n**). The confusion matrix (right) shows the number of correct and incorrect predictions.

The highest balanced accuracy (73.67%) was achieved for the community trusts (table 2). The model had one split based on provider site code, allocating 23 codes a 70% probability of unknown ethnicity (figure 3). The model was effective at predicting unknown ethnicity and discriminating between classes, with a sensitivity of 81.82% and an AUC of 0.737. Provider site code was considered the most important variable, with a relative variable importance of 84. Other variables considered important included the attendance source (5), the number of treatments the patient received (4) and the chief complaint code (3). However, these are not used within the structure of the tree.

**Table 2:** The results from the classification and regression tree (CART) analysis for each organisation type when evaluated on the test set*†‡

	Acute Trusts	Community Trusts	Independent Sector Organisations
**Balanced accuracy**	**63.61%**	**73.67%**	**63.82%**
Sensitivity	65.92%	81.82%	38.89%
AUC	0.636	0.737	0.638

* Applying the 70:15:15 split produced training datasets of 1 512 897 rows (acute trusts), 327 627 rows (community trusts) and 476 145 rows (independent sector organisations).

† A CART model was applied to each dataset via the ‘rpart’ R package.

‡ The 20 variables included in each dataset were as follows: the patient’s age on arrival, the patient’s sex, the Index of Multiple Deprivation (IMD) decile based on the patient’s postcode, the time of arrival (hour of day and day of week), the type of emergency care department (types 1–4), the provider site organisation code, the distance in kilometres between the postcode centroid grid reference for the patient’s address and the postcode centroid grid reference for the provider of the Urgent Emergency Care (UEC) Service, the mode of transport used by the patient to arrive, the source of attendance (eg, walk-in or GP referral), the nature of the patient’s chief complaint on initial assessment, the severity of the patient’s condition on initial assessment (from 1=‘immediate care level emergency care’ to 5=‘low acuity level emergency care’), the time between the patient’s arrival and their initial assessment, the total number of clinical investigations received by the patient, the total number of clinical treatments received by the patient, the total number of patient diagnoses recorded during the attendance, the total number of attendances to an UEC service the patient had between 1 April 2023 and 31 March 2024, the time between the patient’s arrival and departure, the intended destination of the patient following discharge and the target variable (1=’unknown ethnicity’, 0=‘known ethnicity’).

AUC, area under the curve.

**Figure 4:**
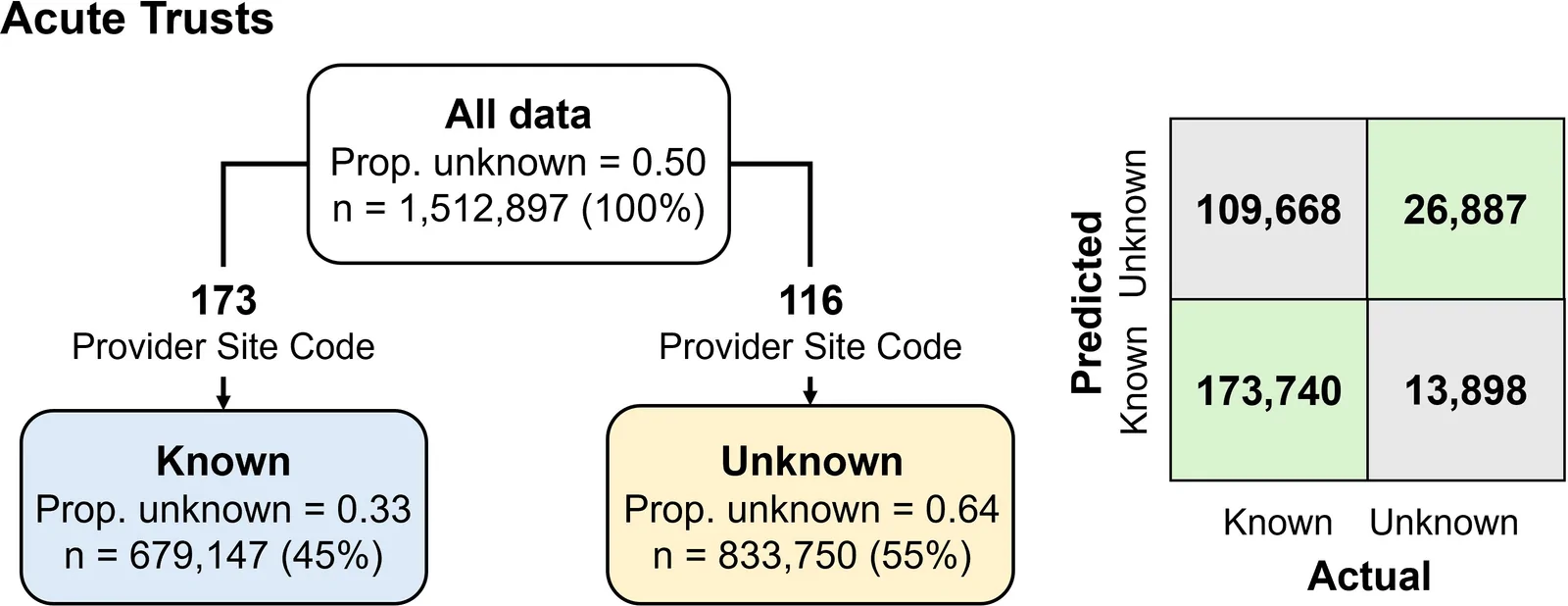
Classification and regression tree (CART) analysis for the acute trusts, showing provider site code as the most important factor in recording ethnicity as unknown. Each node shows the proportion of unknown ethnicity records (prop. unknown) and the total number of records (**n**). The confusion matrix (right) shows the number of correct and incorrect predictions.

For acute trusts, the model achieved a balanced accuracy of 63.61% (table 2). The model only split by provider site code, allocating 116 codes a 64% probability of an unknown ethnicity (figure 4). The model had reasonable ability to discriminate between classes and predict unknown ethnicity, with an AUC of 0.636 and a sensitivity of 65.92%. Provider site code had a relative variable importance of 94. The number of investigations received had a relative variable importance of 3, the source of attendance, chief complaint and department type a relative variable importance of 1.

**Figure 5:**
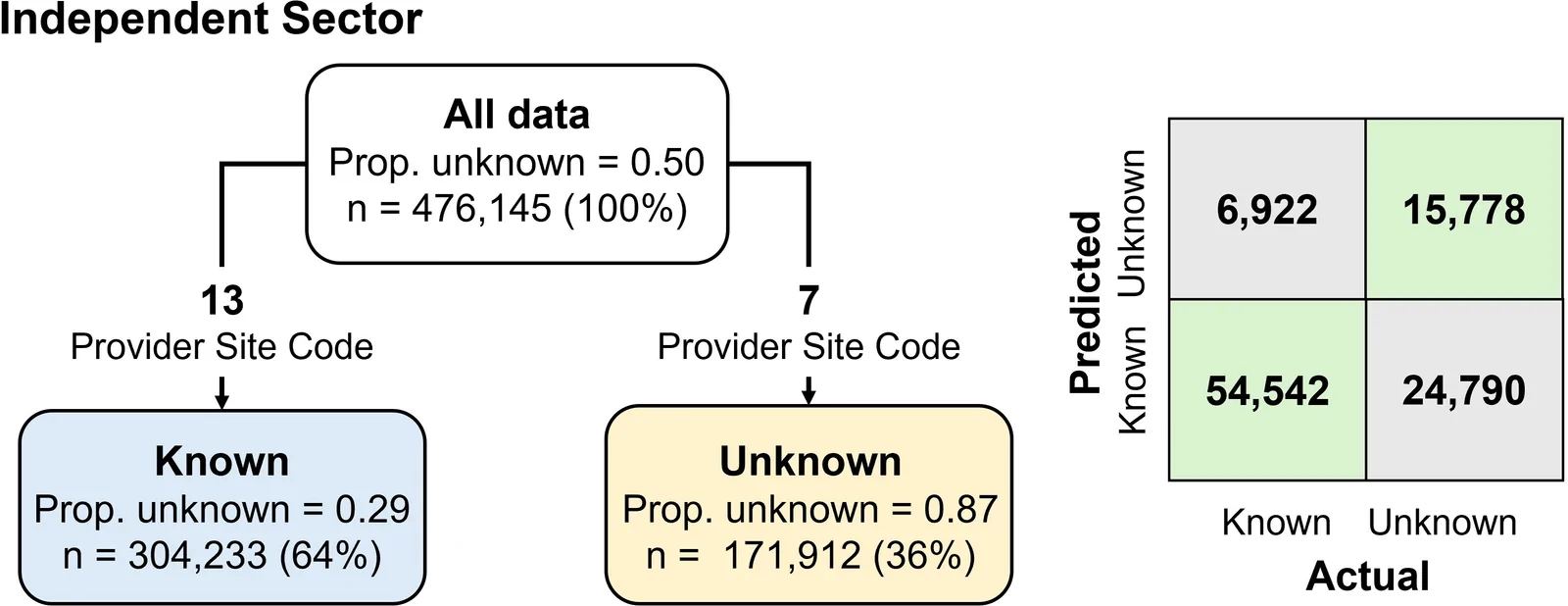
Classification and regression tree (CART) analysis for the independent sector organisations, showing provider site code as the most important factor in recording ethnicity as unknown. Each node shows the proportion of unknown ethnicity records (prop. unknown) and the total number of records (**n**). The confusion matrix (right) shows the number of correct and incorrect predictions.

For the independent sector organisations, the model achieved a balanced accuracy of 63.82% (table 2). As above, the model only split by provider site code, allocating seven codes an 87% probability of unknown ethnicity (figure 5). The model had a reasonable ability to discriminate between classes, with an AUC of 0.638. However, the model demonstrated poor predictive performance, with a sensitivity of 38.89%. Provider site code was considered the most important variable, with a relative variable importance of 78.

All three models highlighted provider site code as the most important variable in recording ethnicity as unknown. Further analysis was carried out by organisation type to explore the variation in ethnicity recording between individual providers in more detail.

**Figure 6:**
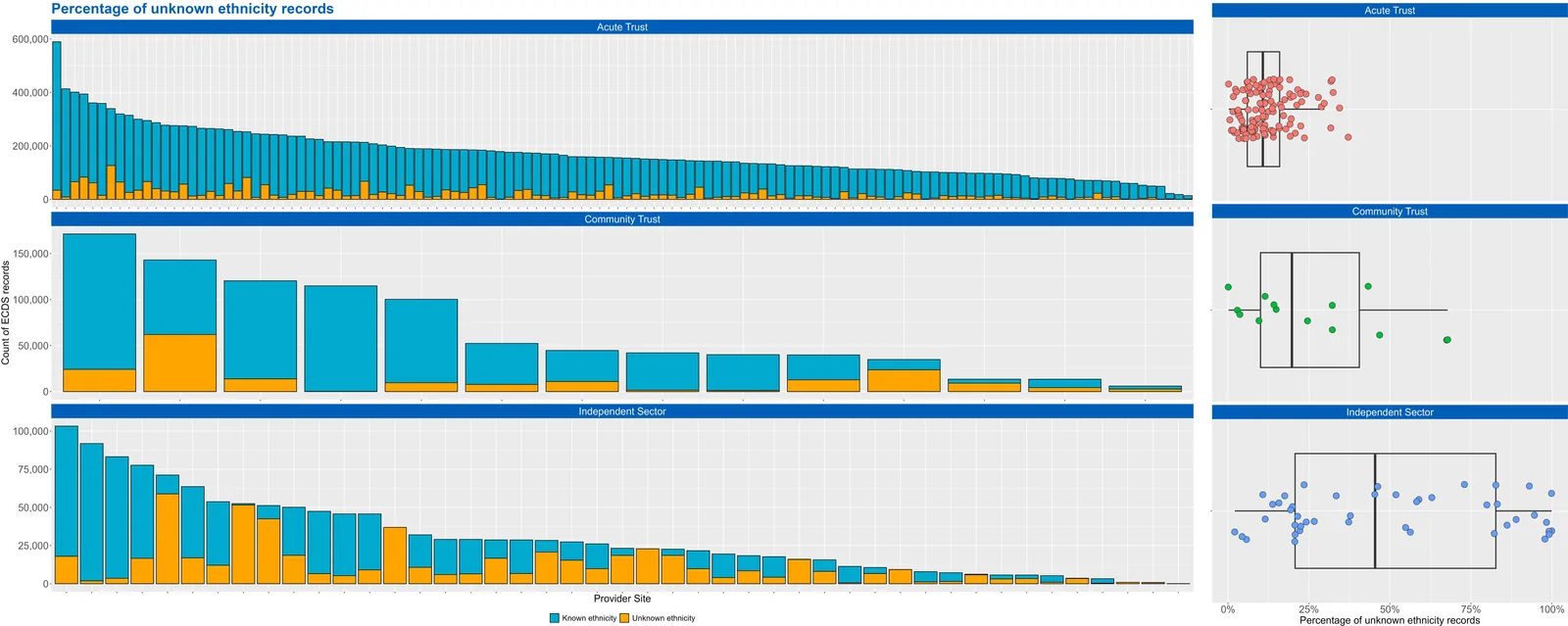
The bar chart shows a count of ECDS records for each organisation by organisation type. The orange bars show the number of ECDS records with an unknown ethnicity, and the blue bars show the number of ECDS records with a known ethnicity. The boxplot shows the range in the percentage of records with an unknown ethnicity for each organisation type. Each dot represents an individual organisation. Independent sector organisations have the largest interquartile range (IQR: 20.70%–82.68%) and the highest median value. Acute trusts have the narrowest interquartile range (IQR: 5.95%–15.94%) and the lowest median value. ECDS, Emergency Care Data Set.

The range in unknown ethnicity recording was smallest for acute trusts (0.24%–37.21%), with most providers below the average percentage of unknown ethnicity records (figure 6). However, there were 35 acute trusts which disproportionately contributed to the total number of unknown ethnicity records. To assess this, a ratio was used to compare the percentage of all unknown ethnicity records attributed to each trust to the percentage of total ECDS records contributed by each trust. 35 trusts had a ratio greater than one, showing they accounted for a greater share of unknown ethnicity records (43.90%) relative to their overall contribution to ECDS records (29.20%). Of these 35 trusts, six had a ratio greater than two, showing they accounted for more than double the percentage of unknown ethnicity records (11.06%) than total ECDS records (4.88%). These findings highlight a subset of acute trusts that were responsible for a disproportionately high number of unknown ethnicity records.

Independent sector organisations had the widest range in the percentage of unknown ethnicity records (2.07%–99.92%), with 36 of the 42 organisations above the average percentage of unknown ethnicity records (figure 6). Eight organisations had more than 90% of records with an unknown ethnicity, equivalent to 4.1% of all unknown ethnicity records (146 463 records). Of these, four organisations had more than 99% of records with an unknown ethnicity, equal to 2.4% of all unknown ethnicity records (84 925 records). These eight organisations represent a higher proportion of unknown ethnicity records (4.1%) compared with total ECDS records (0.6%).

Overall, the CART analysis has demonstrated the importance of provider site code in recording ethnicity as unknown and highlighted a subset of acute trusts and independent sector organisations which disproportionately contributed to unknown ethnicity records.

## Discussion

This study examined unknown ethnicity recording within emergency care data. It has investigated variation in unknown ethnicity records, explored patterns of missingness between ethnicity and other variables and identified the most important variables in recording ethnicity as unknown.

Exploratory analysis demonstrated that 14.91% of ECDS records had an unknown ethnicity, with the category ‘Not Stated’ representing 7.75%, ‘Not Known’ 5.67% and ‘Null’ 1.50%. These proportions are comparable to other recent studies examining the completeness of ethnicity data within ECDS^14 50^ and show an improvement in completeness over time when compared with earlier research.^51^ These proportions also align with existing research examining the completeness of ethnicity data within primary care data^29 52^ and cancer patient experience data.^53^

Variation in unknown ethnicity recording was observed across demographics and attendance characteristics, with younger patients and those requiring low-level emergency care having a higher percentage of unknown ethnicity records. This was also true for patients who spent less time in the department and those with fewer total attendances across the financial year. These findings support the argument that higher levels of healthcare utilisation are associated with increases in completeness of a patient’s electronic healthcare record,^25 29 54^ demonstrating that longer or more frequent interaction with an emergency department increases the likelihood of a valid ethnicity record.

This study also identified 62 variables that saw an increase in the percentage of missing values when ethnicity was unknown (figure 1). The largest increases in missingness were observed in variables related to the investigations and treatments received by the patient, with increases also seen in variables relating to the severity of the patient’s condition, the chief complaint on arrival and place of discharge. Analysis revealed variation across the category of unknown ethnicity, with larger increases in missingness observed when the ethnicity was not asked compared with not declared, and across organisation type.

Combined, these findings highlight challenges within current ethnicity recording processes that might worsen health inequalities. The link between utilisation and ethnicity recording could create representation bias within the data. Ethnic minorities are less likely to engage with healthcare services,^4^ contributing to their under-representation in healthcare data.^16^ This representation bias may be compounded by an increase in missingness across multiple variables when ethnicity is unknown, resulting in further under-representation for those without a known ethnicity. It follows that complete and accurate ethnicity data are necessary to mitigate bias caused by missing data,^22 25 54^ which could entrench existing health inequalities if unaddressed.

The CART models identified provider site code as the most important variable in recording ethnicity as unknown. Further analysis identified a subset of acute trusts and independent sector organisations which disproportionately contributed to unknown ethnicity records. This is consistent with previous research, where logistic regression was used to understand the predictors of missing ethnicity.^53^ The authors concluded the data collection processes of individual providers were the primary driver, evidenced by large variation in ethnicity recording across individual sites.^53^ Moreover, qualitative studies have demonstrated that ethnicity recording is likely influenced by the data collection process; poor data quality has been linked to staff reluctance to ask for a patient’s ethnicity,^18^ a limited understanding of why ethnicity data should be collected^55^ and challenges in collection caused by the pressured clinical setting.^55^ These are actionable insights regarding the data collection process that can be used to aid the improvement of ethnicity recording.

### Strengths and limitations

This study uses data visualisation and machine learning techniques to better understand ethnicity recording within emergency care data. It is the first application of CART analysis on the ECDS and successfully identifies the most important variable in recording ethnicity as unknown, while achieving an accuracy comparable to the 69%–85% achieved in previous studies.^33^ Similarly, it is the first time advanced data visualisation techniques have been applied to the ECDS to uncover patterns within missing data. These methods demonstrated increases in missingness across several variables when ethnicity was unknown and highlighted key differences in the structure of missingness between organisation types and the different categories of unknown ethnicity.

Previous studies have aggregated the ECDS within Hospital Episode Statistics to analyse the quality of ethnicity data,^14 50^ therefore combining emergency care data with inpatient and outpatient datasets. By focusing on the ECDS, this study demonstrated variation in ethnicity recording across characteristics related specifically to an A&E attendance, such as the severity of the patient’s condition on arrival, the time spent in the department and the number of attendances within the financial year.

The methods used in this study could be generalised to other datasets and missing variables. The CART models are easy to understand,^33^ and the data visualisation techniques offer accessible interpretation of complex missing data structures.^31 56^ Future work could apply these techniques across NHS datasets and to variables other than ethnicity. This would increase understanding of missing data and data collection processes across healthcare.

The study had two key limitations. First, the performance of the CART models could be improved. Accuracy was lowest for acute trusts (table 2). Since acute trusts account for the majority of ECDS records (table 1), increased accuracy would be beneficial. Several factors may influence model accuracy: (a) categorical variables were used to reduce run-time and to use the surrogate methodology for missing data offered by the CART model. However, this meant values within the validation and test sets had to be matched to those in the training data, which may have limited accuracy. (b) Variables were selected based on the exploratory analysis and conversations with domain experts. Future work could use more advanced techniques to aid variable selection, such as principal component analysis, which may increase accuracy. (c) It is recognised that unknown ethnicity recording may be influenced by factors not captured within the ECDS. Although the analysis included variables previously shown to be associated with missingness—such as healthcare utilisation^25 29 54^ —there may be additional influences absent from the ECDS that affect the likelihood of unknown ethnicity being recorded and, therefore, the accuracy of the model.

Second, while the study focused on the completeness of ethnicity recording, it did not consider the accuracy of the data. Research has shown patients from an ethnic minority background are more likely to have multiple ethnicities recorded than patients from a white background.^16^ There is also lower agreement for ethnic minorities between the ethnicity recorded in the ECDS and the 2021 Census^57^ and other NHS datasets.^53 58^ Accuracy is crucial to ensure quality ethnicity data, and therefore, future work should consider if the factors most important in completeness also influence accuracy.

## Conclusion

This study has improved understanding of ethnicity recording in emergency care data by exploring variation in unknown ethnicity records, demonstrating an increase in missingness in other variables when ethnicity is unknown and identifying provider site code as the most important variable in recording an unknown ethnicity. These insights have been shared with NHS England alongside recommendations for future work, focused on improving data collection processes for the individual organisations with a disproportionately high number of unknown ethnicity records. This would provide direct benefits for NHS England and NHS providers and is necessary for tackling health inequalities.

## Supplementary material

10.1136/bmjopen-2026-118200 online supplemental file 1

## Data Availability

Data may be obtained from a third party and are not publicly available.
