# Supplementary material for: Using classification and regression trees (CART) to investigate unknown ethnicity records in NHS emergency care data
Source: BMJ Open. 2026 Aug 17;16(8):e118200. doi: 10.1136/bmjopen-2026-118200 (PMC13504918; doi:10.1136/bmjopen-2026-118200)
Supplement: Supplementary file 1 [file bmjopen-2026-118200.reviewer_comments.pdf]

PEER REVIEW HISTORY

BMJ Open publishes all reviews undertaken for accepted manuscripts. Reviewers are asked to complete a checklist review form and are provided with free text boxes to elaborate on their assessment. These free text comments are reproduced below.

ARTICLE DETAILS

Title (Provisional)

Using classification and regression trees (CART) to investigate unknown ethnicity records in NHS emergency care data

Authors

Dean, Grace; Ruddle, Roy A

VERSION 1 - REVIEW

**Reviewer**	**1**
**Name**	**Dack, Kyle**
**Affiliation**	**University of Bristol, MRC Integrative Epidemiology Unit**
**Date**	**16-Apr-2026**
**COI**	**None**

Statistical review summary:

Overall the paper is methodologically strong, and the reporting is clear and aligned with TRIPOD. However, the reporting of variables could be made clearer. In particular: (1) Explaining the types of variables included, because NHS codes will not be understandable to all readers. (2) Explaining the criteria for passing variables through the analysis, and (3) adding specifics to the results about which variables were modelled and their relative importance. The discussion could also consider the possibility of unobserved factors.

Abstract:

1. The N is reported (24,167,154) but the current placement makes it look like it is the total number of exclusions. "24,167,154 records after exclusions" (or similar) would avoid this.

Methods:

2. Data source: records with missing pseudo-NHS number were excluded, I suggest adding a reason for this, e.g. if it precluded linkage to the relevant data.

3. Predictors: The outcome - ethnicity missingness, was defined, but the predictors are not summarized in the main text. While variables are listed in Table S2 and S3, a brief in-text description of the types of variables included, how they were selected, and reference to any

information about measurement methods would improve the transparency and align with TRIPOD item 7a. This is particularly important for those variables which were dropped, which are only reported as NHS codes and may not be interpretable by all readers.

4. " Second, the structure of missing data within ECDS was analysed" I suggest clarifying that this means missing data for variables other than ethnicity.

5. The core statistical methodology, including CART models, is reported clearly and methodologically sound. However, the criteria used to include variables could be described more explicitly: were there any pre-defined or data-driven statistical thresholds required? How was this integrated with input from domain experts? This is important for the interpretation because it clarifies how the model reflects prior knowledge and present data signals.

Results:

6. For readers unfamiliar with CART or without a statistical background, the authors could add a brief sentence for what balanced accuracy represents and its intended interpretation.

7. Table 2. I suggest adding as a footnote which variables this is based upon, the N, and statistical model used. Variables are listed in supplementary table S3 but this is not commented on in the results, and is important to the interpretation. Any further specifics about how the variables were selected would also improve the clarity of the analysis.

8. The relative importance of a few variables is reported, but are the full model results available? Providing the full list in a supplementary table (or appending to table S3) could improve the transparency of this part of the analysis.

Discussion:

9. The interpretation is reasonable and I have no major concerns. However, there is one potential limitation which could be added. The analysis is based upon observed factors, but there is always a risk of unmeasured factors influencing the missingness of ethnicity. Missing data could be due to latent factors which are not captured in the data, and by definition this is not statistically testable (i.e. the MNAR problem). It would be helpful for readers if the authors could confirm that all theoretically relevant factors were mapped to NHS variables, or if there are any factors with evidence of links to missingness in prior studies which were omitted in this analysis.

Reviewer 2

Name Trent, Stacy

Affiliation Denver Health Medical Center, Department of Emergency Medicine

Date 27-Apr-2026

**COI**	**None**

Thank you for working in this space. This is an interesting look at ethnicity data in the UK and is highly relevant for equality/equity work. Coming from the US perspective, this is highly surprising that there is this much missingness and variability in missingness in this data. The categories of these variables are also seemingly discordant. If null is not entered, not known is not asked and not stated is asked and not answered, the associated variables with missingness do not seem rational. Why is "not stated" so common among young people with low acuity problems. They can certainly answer the question. Are they really not answering the question or is the question not being asked which seems more likely in this population. My other question is are these patients being seen, particularly as clinically relevant variables (diagnosis and treatments) seem missing. Is the emergency care data linked to other NHS datasets? Can ethnicity be identified that way? Is ethnicity not linked to the patient's NHS # (if that exists?)? In a single payer / government run health system, it seems redundant to ask these sorts of questions on every visit. Are these not asked at enrollment and carried forward in perpetuity? At a high level, I would add some discussion about more efficient ways to capture and link this data in a system like yours.

Couple small comments - the variable names in the figures are hard to interpret. They are listed in table 2, but there is no context to what these mean. They maybe described by NHS, but that is a leap to ask someone to go search for this. Maybe remove the R variable name and replace with what it means (example: "RTT_Period_Status" - what does RTT mean etc?), especially in the published figures

VERSION 1 - AUTHOR RESPONSE

Reviewer 1

Overall the paper is methodologically strong, and the reporting is clear and aligned with TRIPOD. However, the reporting of variables could be made clearer. In particular: (1) Explaining the types of variables included, because NHS codes will not be understandable to all readers. (2) Explaining the criteria for passing variables through the analysis, and (3) adding specifics to the results about which variables were modelled and their relative importance. The discussion could also consider the possibility of unobserved factors.

Thank you for your comments. Comment (1) and (2) in the above are addressed by the changes described in response 3 below. Comment (3) is addressed by the changes described in responses 8 and 9.

Abstract:

1. The N is reported (24,167,154) but the current placement makes it look like it is the total number of exclusions. "24,167,154 records after exclusions" (or similar) would avoid this.

We have added "after exclusions" as suggested.

Methods:

2. Data source: records with missing pseudo-NHS number were excluded, I suggest adding a reason for this, e.g. if it precluded linkage to the relevant data.
   In Methods -> Data we have added an explanation that rows without an anonymised pseudo-NHS number cannot be validated, deduplicated or reliably linked.

3. Predictors: The outcome - ethnicity missingness, was defined, but the predictors are not summarized in the main text. While variables are listed in Table S2 and S3, a brief in-text description of the types of variables included, how they were selected, and reference to any information about measurement methods would improve the transparency and align with TRIPOD item 7a. This is particularly important for those variables which were dropped, which are only reported as NHS codes and may not be interpretable by all readers.
   We have added a plain English name and variable description to Supplementary Table 2 and Supplementary Table 3 to increase interpretability. We have also added an in-text description of the types of variables included and a brief explanation why variables were excluded from the analysis (see "The variables were chosen to ensure the model would remain interpretable to NHS England and provider staff …" in Methods -> Analysis).

4. "Second, the structure of missing data within ECDS was analysed" I suggest clarifying that this means missing data for variables other than ethnicity.
   We have changed the sentence to "Second, the structure of missing data for all ECDS variables other than ethnicity was analysed" for clarification (see Methods -> Analysis).

5. The core statistical methodology, including CART models, is reported clearly and methodologically sound. However, the criteria used to include variables could be described more explicitly: were there any pre-defined or data-driven statistical thresholds required? How was this integrated with input from domain experts? This is important for the interpretation because it clarifies how the model reflects prior knowledge and present data signals.
   This is addressed by the changes we made for comment 3 - we have added an in-text description of the types of variables included and a brief explanation why variables were excluded from the analysis.

Results:

6. For readers unfamiliar with CART or without a statistical background, the authors could add a brief sentence for what balanced accuracy represents and its intended interpretation.
   We have added a paragraph that explains balanced accuracy towards the beginning of the results section ("The primary evaluation metric was balanced accuracy …"; just below Table 2).

7. Table 2. I suggest adding as a footnote which variables this is based upon, the N, and statistical model used. Variables are listed in supplementary table S3 but this is not commented on in the results, and is important to the interpretation. Any further specifics about how the variables were selected would also improve the clarity of the analysis.
   We have added three footnotes below Table 2 which includes the size of the training datasets, the statistical model used (CART) and the variables included in the model.

8. The relative importance of a few variables is reported, but are the full model results available? Providing the full list in a supplementary table (or appending to table S3) could improve the transparency of this part of the analysis.
   We have listed all variables and their relative importance for each of the organisation types (Supplementary Table 5).

Discussion:

9. The interpretation is reasonable and I have no major concerns. However, there is one potential limitation which could be added. The analysis is based upon observed factors, but there is always a risk of unmeasured factors influencing the missingness of ethnicity. Missing data could be due to latent factors which are not captured in the data, and by definition this is not statistically testable (i.e. the MNAR problem). It would be helpful for readers if the authors could confirm that all theoretically relevant factors were mapped to NHS variables, or if there are any factors with evidence of links to missingness in prior studies which were omitted in this analysis.
   Many thanks for highlighting this, a very important point to raise. Agree that whilst we included variables previously shown to be associated with missingness (such as the total number of A&E attendances as an indicator of previous healthcare utilisation), there may be additional influences absent from ECDS that affect the likelihood of unknown ethnicity being recorded. We have included this within the Strengths and Limitations section.

Reviewer 2

Dr. Stacy Trent, Denver Health Medical Center, University of Colorado Denver School of Medicine

Comments to the Author:

Thank you for working in this space. This is an interesting look at ethnicity data in the UK and is highly relevant for equality/equity work. Coming from the US perspective, this is highly surprising that there is this much missingness and variability in missingness in this data. The categories of these variables are also seemingly discordant. If null is not entered, not known is not asked and not stated is asked and not answered, the associated variables with missingness do not seem rational. Why is "not stated" so common among young people with low acuity problems. They can certainly answer the question. Are they really not answering the question or is the question not being asked which seems more likely in this population.

Thank you for your comments. The analysis showed that younger patients and those presenting with lower-acuity conditions had higher overall levels of unknown ethnicity recording. However, it did not separately examine the proportions of "Not Stated", "Not Known", and "Null" within each demographic or attendance group. Future work could explore how these individual categories vary for younger patients and low-acuity attendances, but based on the current analysis, it is not possible to conclude that "Not Stated" is particularly common among young people with low-acuity problems.

My other question is are these patients being seen, particularly as clinically relevant variables (diagnosis and treatments) seem missing. Is the emergency care data linked to other NHS datasets? Can ethnicity be identified that way? Is ethnicity not linked to the patient's NHS # (if that exists?)? In a single payer / government run health system, it seems redundant to ask these sorts of questions on every visit. Are these not asked at enrollment and carried forward in perpetuity? At a high level, I would add some discussion about more efficient ways to capture and link this data in a system like yours.

Work is currently ongoing within the NHS to introduce the Single Patient Record (SPR). This will link patient data (including ethnicity data) from multiple NHS datasets by 2028 and should improve the quality of ethnicity data and reduce administrative burden. However, this analysis is still relevant as it demonstrates the importance of the data collection processes for individual providers on data quality and the variation across the system, key factors to consider when linking data to create the SPR.

Couple small comments - the variable names in the figures are hard to interpret. They are listed in table 2, but there is no context to what these mean. They maybe described by NHS, but that is a leap to ask someone to go search for this. Maybe remove the R variable name and replace with what it means (example: "RTT_Period_Status" - what does RTT mean etc?), especially in the published figures

We have updated the variable names in Figures 1 - 5 to be a plain English description (for example, "Referral to Treatment Status" rather than "RTT_Period_Status"). We have also added the plain English name and variable description to Supplementary Table 2 and Supplementary Table 3 to increase interpretability.
