# Supplementary material for: Using classification and regression trees (CART) to investigate unknown ethnicity records in NHS emergency care data
Source: BMJ Open. 2026 Aug 17;16(8):e118200. doi: 10.1136/bmjopen-2026-118200 (PMC13504918; doi:10.1136/bmjopen-2026-118200)
Supplement: Supplementary file 2 [file bmjopen-16-8-s001.docx]

## **Using classification and regression trees (CART) to investigate unknown ethnicity records in NHS emergency care data**

Grace Dean ^1^, Roy A Ruddle ^2^

^1^ NHS England

^2^ School of Computing and Leeds Institute for Data Analytics, University of Leeds, Leeds, UK

## **Supplementary Material**

**Tables**

**Table 1: The definition of each ethnic code used in ECDS, as described (NHS England, 2025). Definitions align with the 2001 Census definitions (Cabinet Office, 2024).**

| **Code** | **Ethnic Category** | **Ethnicity** |
| --- | --- | --- |
| A | White | British |
| B | White | Irish |
| C | White | Any other White background |
| D | Mixed | White and Black Caribbean |
| E | Mixed | White and Black African |
| F | Mixed | White and Asian |
| G | Mixed | Any other mixed background |
| H | Asian or Asian British | Indian |
| J | Asian or Asian British | Pakistani |
| K | Asian or Asian British | Bangladeshi |
| L | Asian or Asian British | Any other Asian background |
| M | Black or Black British | Caribbean |
| N | Black or Black British | African |
| P | Black or Black British | Any other Black background |
| R | Other Ethnic Groups | Chinese |
| S | Other Ethnic Groups | Any other ethnic group |
| Z | Not stated | Not stated |
| 99 | Not known | Not known |

**Table 2: The 208 ECDS variables used during analysis. Variable names as described**

**(NHS England, 2025).**

| **Variable** | **Variable Name** | **Definition** |
| --- | --- | --- |
| Emergency Care Record ID | EC_Ident | A unique identifier assigned to each Emergency Care attendance record. |
| Reporting Period Start Date | Report_Period_Start_Date | The start date of the reporting period. |
| Reporting Period End Date | Report_Period_End_Date | The end date of the reporting period. |
| Provider Organisation Code | Provider_Code | The organisation code of the healthcare provider submitting the record. |
| Commissioner Organisation Code | Commissioner_Code | The organisation code of the commissioner responsible for funding the patient’s care. |
| Converted Booking Reference | Unique_Booking_Reference_Number_Converted | A converted version of the Unique Booking Reference Number (UBRN) used to track referral‑to‑treatment pathways. |
| UBRN Issuing Organisation | UBRNIssuer | The organisation responsible for issuing the UBRN. |
| Pathway ID Issuer | Org_Code_Patient_Pathway_ID_Issuer | The organisation that issued the Patient Pathway Identifier for RTT tracking. |
| Referral to Treatment (RTT) Status | RTT_Period_Status | Indicates if the patient is currently on a RTT pathway and their status. |
| Waiting Time Measurement Type | Wait_Time_Measurement_Type | Indicates the method used to calculate the patient’s waiting time (e.g., includes consultation-led waiting time). |
| RTT Start Date | RTT_Period_Start_Date | The date the patient’s RTT period began. |
| RTT End Date | RTT_Period_End_Date | The date the patient’s RTT period ended. |
| RTT Duration | RTT_Period_Length | The total number of days in the RTT period. |
| NHS Number Status Indicator | NHS_Number_Status_Indicator_Code | A code indicating the validity or verification status of the NHS number. |
| NHS Number Valid Flag | NHS_Number_Is_Valid | A flag showing whether the NHS number passed validation. |
| Local Patient Identifier | Local_Patient_ID | A patient identifier assigned locally by the treating organisation. |
| Local Patient ID Issuer | Org_Code_Local_Patient_ID | The organisation that issued the local patient identifier. |
| Identity Withheld Reason | Withheld_Identity_Reason | The reason why patient identity details were withheld or not provided. |
| Birth Month | Month_of_Birth | The month the patient was born. |
| Birth Year | Year_of_Birth | The year the patient was born. |
| **Variable** | **Variable Name** | **Definition** |
| Patient Administrative Type | Patient_Type | Indicates whether the patient is new, unregistered, or of another administrative type. |
| Age at Activity Date | Age_at_CDS_Activity_Date | The patient’s age in complete years on the date the CDS activity occurred. |
| Age at Arrival | Age_At_Arrival | The age of the patient on date the activity took place. Derived as the number of complete years between the patient’s birth date and the activity date. |
| Fractional Age at Arrival | Fractional_Age_At_Arrival | The patient’s age at arrival expressed in years with decimal precision. |
| HES Age at Arrival | HES_Age_At_Arrival | The patient’s age at arrival as derived for Hospital Episode Statistics. |
| HES Age at Departure | HES_Age_At_Departure | The patient’s age at departure as derived for Hospital Episode Statistics. |
| Postcode District | Postcode_District | The outward portion of the patient’s postcode, used for geographic grouping and analysis. |
| 2011 Output Area | 2011_Output_Area | The 2011 Census Output Area associated with the patient’s postcode. |
| 2011 MSOA | 2011_MS0A | The 2011 Census Middle Layer Super Output Area linked to the patient’s postcode. |
| 2011 LSOA | 2011_LSOA | The 2011 Census Lower Layer Super Output Area linked to the patient’s postcode. |
| Country of Residence | Country | The country in which the patient normally resides. |
| Government Office Region | Government_Office_Region | The Government Office Region associated with the patient’s postcode. |
| Residence County | Residence_County | The county in which the patient resides, derived from their postcode. |
| Local Authority District | Local_Authority_District | The local authority district corresponding to the patient’s postcode. |
| Parliamentary Constituency | Parliamentary_Constituency | The UK parliamentary constituency covering the patient’s postcode. |
| Electoral Division County Code | ED_County_Code | The county electoral division code associated with the patient’s postcode. |
| Electoral Division District Code | ED_District_Code | The district electoral division code linked to the patient’s postcode. |
| Electoral Ward (1998 boundaries) | Electoral_Ward_1998 | The electoral ward of residence based on 1998 boundary definitions. |
| **Variable** | **Variable Name** | **Definition** |
| IMD Decile | Index_Of_Multiple_Deprivation_Decile | A measure of relative deprivation based on where the patient resides. Areas are ranked based on their overall deprivation score and divided into deciles (1 = “Most deprived areas”, 10 = “Least deprived areas”). |
| IMD Decile Description | Index_Of_Multiple_Deprivation_Decile_Description | A textual description of the patient’s deprivation decile category. |
| Rural/Urban Indicator | Rural_Urban_Indicator | Indicates whether the patient’s postcode is classified as rural or urban. |
| Cancer Registry Region | Cancer_Registry | The cancer registry region associated with the patient’s postcode. |
| Grid Reference Link | Grid_Link_Reference | A geographic grid reference derived from the patient’s postcode. |
| Residence Responsibility Organisation | Org_Code_Residence_Responsibility | The organisation responsible for commissioning care based on the patient’s residence. |
| Organisation from Postcode | Organisation_Id_From_Patient_Postcode | The organisation code derived directly from the patient’s postcode. |
| HES CCG from Postcode | HES_CCG_From_Patient_Postcode | The Clinical Commissioning Group assigned to the patient based on postcode for HES reporting. |
| Sex | Sex | The person phenotypic sex of the patient (1 = “Male”, 2 = “Female”,  9 = “Indeterminate”, X = “Not Known”). |
| Ethnic Category | Ethnic_Category | The patient’s ethnicity coded using the ONS 2001 classification. |
| Accommodation Status (SNOMED) | Accommodation_Status_SNOMED_CT | The SNOMED CT code describing the patient’s accommodation status. |
| Accommodation Status (Approved Code) | Accommodation_Status_Code_Approved | The approved national code for the patient’s accommodation status. |
| Preferred Spoken Language (SNOMED) | Preferred_Spoken_Language_SNOMED_CT | The SNOMED CT code representing the patient’s preferred spoken language. |
| Preferred Spoken Language (Approved Code) | Preferred_Spoken_Language_Code_Approved | The approved national code for the patient’s preferred spoken language. |
| Accessible Information Professional Required (SNOMED) | Accessible_Information_Professional_Required_SNOMED_CT | The SNOMED CT code indicating whether a professional is required to support accessible communication. |
| **Variable** | **Variable Name** | **Definition** |
| Accessible Information Professional Required (Approved Code) | Accessible_Information_Professional_Required_Code_Approved | The approved national code indicating whether accessible communication support is required. |
| Interpreter Language (SNOMED) | Interpreter_Language_SNOMED_CT | The SNOMED CT code representing the language required for interpretation. |
| Interpreter Language (Approved Code) | Interpreter_Language_Code_Approved | The approved national code for the interpreter language required. |
| Overseas Visitor Charging Category | Overseas_Visitor_Charging_Category | Indicates the patient’s charging category for overseas visitor status. |
| GP Code | GP_Code | The code identifying the patient’s registered GP. |
| GP Practice Code | GP_Practice_Code | The code identifying the patient’s registered GP practice. |
| PDS General Practice Code | PDS_General_Practice_Code | The GP practice code recorded in the Personal Demographics Service. |
| Responsible CCG (from GP) | Responsible_CCG_From_General_Practice | The CCG responsible for the patient based on their GP practice. |
| HES CCG (from GP) | HES_CCG_From_General_Practice | The HES‑derived CCG based on the patient’s GP practice. |
| HES Responsible CCG Value | HES_Responsible_CCG_Value | The CCG value assigned for HES reporting. |
| HES Responsible CCG Origin | HES_Responsible_CCG_Origin | The method used to derive the HES responsible CCG. |
| Attendance Unique Identifier | Attendance_Unique_Identifier | A unique identifier assigned to each Emergency Care attendance. |
| Treatment Site Code | Site_Code_of_Treatment | The organisation site code where the patient received emergency care. |
| Attendance Postcode District | Attendance_Postcode_District | The postcode district associated with the location of attendance. |
| Attendance Government Office Region | Attendance_Government_Office_Region | The Government Office Region linked to the attendance location. |
| Attendance SHA Provider | Attendance_SHA_Provider | The Strategic Health Authority responsible for the provider site. |
| Attendance HES CCG (Treatment Site Code) | Attendance_HES_CCG_From_Treatment_Site_Code | The HES‑derived CCG based on the treatment site code. |
| Attendance HES CCG Origin | Attendance_HES_CCG_From_Treatment_Origin | The method used to derive the HES CCG for the treatment site. |
| **Variable** | **Variable Name** | **Definition** |
| Emergency Department Type | EC_Department_Type | The type of emergency care department (01 = “Major Emergency Care Department”, 02 = “Mono-specialty Emergency Care Department”,  03 = “Urgent Treatment Centre”,  04 = “NHS Walk-In Centre”). |
| Distance: LSOA to Provider | Attendance_LSOA_Provider_Distance | The distance between the patient’s LSOA of residence and the provider site. |
| Distance Origin: LSOA to Provider | Attendance_LSOA_Provider_Distance_Origin | The method used to calculate the LSOA‑to‑provider distance. |
| Distance: LSOA to Treatment Site | Attendance_LSOA_Treatment_Site_Distance | The distance between the patient’s LSOA and the specific treatment site. |
| Distance Origin: LSOA to Treatment Site | Attendance_LSOA_Treatment_Site_Distance_Origin | The method used to calculate the LSOA‑to‑treatment‑site distance. |
| Attendance Number | EC_Attendance_Number | A sequential number identifying the patient’s attendance within the reporting period. |
| Arrival Date | Arrival_Date | The date the patient arrived. |
| Arrival Time | Arrival_Time | The time of day the patient arrived. |
| Arrival Month | Arrival_Month | The month the patient arrived. |
| Arrival Mode (SNOMED) | EC_Arrival_Mode_SNOMED_CT | The SNOMED CT describing the mode of transport the patient used to arrive (e.g., ambulance or walk-in). |
| Arrival Mode (Approved Code) | Arrival_Mode_Approved | The approved national code representing the patient’s arrival mode. |
| Attendance Category | EC_AttendanceCategory | Indicates whether the attendance was unplanned, planned, or another category. |
| Arrival Planned Flag | Arrival_Planned | Indicates whether the patient’s arrival was planned rather than unscheduled. |
| Ambulance Incident Number | Ambulance_Incident_Number | The incident number assigned by the ambulance service for the episode. |
| Ambulance Trust Code | Org_Code_Ambulance_Trust | The organisation code of the ambulance trust involved in the patient’s care. |
| Attendance Source (SNOMED) | EC_Attendance_Source_SNOMED_CT | The SNOMED CT code describing the source from which the patient was referred or arrived. |
| Attendance Source (Approved Code) | Attendance_Source_Approved | The approved national code representing the patient’s attendance source. |
| Attendance Source Organisation | Attendance_Source_Organisation | The organisation responsible for referring or sending the patient to the Emergency Department. |
| **Variable** | **Variable Name** | **Definition** |
| Initial Assessment Date | EC_Initial_Assessment_Date | The date on which the patient first received a clinical assessment after arrival. |
| Initial Assessment Time | EC_Initial_Assessment_Time | The time at which the patient first received a clinical assessment. |
| Time to Initial Assessment | EC_Initial_Assessment_Time_Since_Arrival | The time between the patient’s arrival and their initial clinical assessment. |
| Seen for Treatment Date | EC_Seen_For_Treatment_Date | The date on which the patient was first seen by a clinician for treatment. |
| Seen for Treatment Time | EC_Seen_For_Treatment_Time | The time at which the patient was first seen by a clinician for treatment. |
| Time to Treatment | EC_Seen_For_Treatment_Time_Since_Arrival | The time between arrival and the patient being seen for treatment. |
| Clinical Conclusion Date | EC_Conclusion_Date | The date the clinical episode reached its conclusion. |
| Clinical Conclusion Time | EC_Conclusion_Time | The time the clinical episode reached its conclusion. |
| Time to Clinical Conclusion | EC_Conclusion_Time_Since_Arrival | The time between arrival and the clinical conclusion. |
| Departure Date | EC_Departure_Date | The date the patient left the Emergency Department. |
| Departure Time | EC_Departure_Time | The time the patient left the Emergency Department. |
| Time to Departure | EC_Departure_Time_Since_Arrival | The time between the patient’s arrival and departure. |
| Decision to Admit Date | EC_Decision_To_Admit_Date | The date a clinician decided the patient required admission. |
| Decision to Admit Time | EC_Decision_To_Admit_Time | The time a clinician decided the patient required admission. |
| Time to Decision to Admit | EC_Decision_To_Admit_Time_Since_Arrival | The time between the patient’s arrival and the decision to admit. |
| Decision to Admit Treatment Function | Decision_To_Admit_Treatment_Function_Code | The treatment function code representing the specialty responsible for the admission decision. |
| Receiving Site for Admission | Decision_To_Admit_Receiving_Site | The organisation site where the patient is expected to be admitted following the decision. |
| Discharge Status (SNOMED) | EC_Discharge_Status_SNOMED_CT | The SNOMED CT code describing the patient’s clinical status at discharge. |
| Discharge Status (Approved Code) | EC_Discharge_Status_Approved | The approved national code representing the patient’s discharge status. |
| Discharge Destination (SNOMED) | Discharge_Destination_SNOMED_CT | The SNOMED CT code describing the intended destination of the patient following discharge. |
| **Variable** | **Variable Name** | **Definition** |
| Discharge Destination (Approved Code) | DischargeDestinationApproved | The approved national code for the patient’s discharge destination. |
| Discharge Follow‑Up (SNOMED) | Discharge_Follow_Up_SNOMED_CT | The SNOMED CT code describing the follow‑up arrangements after discharge. |
| Discharge Follow‑Up (Approved Code) | Discharge_Follow_Up_Approved | The approved national code for follow‑up arrangements after discharge. |
| Discharge Information Given (SNOMED) | Discharge_Information_Given_SNOMED_CT | The SNOMED CT code describing the information provided to the patient at discharge. |
| Discharge Information Given (Approved Code) | Discharge_Information_Given_Approved | The approved national code for discharge information provided. |
| Chief Complaint (SNOMED) | EC_Chief_Complaint_SNOMED_CT | The SNOMED CT code describing the patient’s main presenting complaint. |
| Acuity (SNOMED) | EC_Acuity_SNOMED_CT | The SNOMED CT code describing the severity of the patient’s condition at the time of initial assessment at the Urgent and Emergency Care Service.  (1 = “Immediate care level emergency care”,  2 = “Very urgent level emergency care”,  3 = “Urgent level emergency care”  4 = “Standard level emergency care”  5 = “Low acuity level emergency care”). |
| Injury Date | EC_Injury_Date | The date the patient’s injury occurred. |
| Injury Time | EC_Injury_Time | The time the patient’s injury occurred. |
| Injury Activity Status (SNOMED) | EC_Injury_Activity_Status_SNOMED_CT | The SNOMED CT code describing the patient’s activity status at the time of injury. |
| Injury Activity Type (SNOMED) | EC_Injury_Activity_Type_SNOMED_CT | The SNOMED CT code describing the type of activity the patient was engaged in when injured. |
| Injury Intent (SNOMED) | EC_Injury_Intent_SNOMED_CT | The SNOMED CT code describing whether the injury was accidental, deliberate, or of unknown intent. |
| Injury Mechanism (SNOMED) | EC_Injury_Mechanism_SNOMED_CT | The SNOMED CT code describing the mechanism that caused the injury. |
| Place of Injury (SNOMED) | EC_Place_Of_Injury_SNOMED_CT | The SNOMED CT code describing where the injury occurred. |
| Injury Location Latitude | EC_Place_Of_Injury_Latitude | The latitude coordinate of the location where the injury occurred. |
| **Variable** | **Variable Name** | **Definition** |
| Injury Location Longitude | EC_Place_Of_Injury_Longitude | The longitude coordinate of the location where the injury occurred. |
| Clinical Trial Code | Clinical_Trial_Code | A code indicating whether the patient is enrolled in a clinical trial. |
| Disease Notification Flag | Clinical_Disease_Notification | Indicates whether the patient’s condition requires statutory disease notification. |
| Service Agreement Provider | Service_Agreement_Provider | The provider organisation associated with the service agreement for the attendance. |
| Service Agreement Commissioner | Service_Agreement_Commissioner | The commissioner organisation associated with the service agreement. |
| Commissioning Serial Number | Commissioning_Serial_Number | A serial number used to identify the commissioning arrangement. |
| Agreement Line Number | Agreement_Line_Number | The line number within the commissioning agreement relevant to the attendance. |
| Provider Reference Number | Provider_Reference_Number | A provider‑assigned reference number for the attendance. |
| Commissioner Reference Number | Commissioner_Reference_Number | A commissioner‑assigned reference number for the attendance. |
| SHA Commissioner | SHA_Commissioner | The Strategic Health Authority responsible for commissioning the care. |
| Commissioner Recognised Flag | Is_Commissioner_Recognised | Indicates whether the commissioner is recognised as valid for reporting. |
| Commissioning Region | Commissioning_Government_Office_Region | The Government Office Region associated with the commissioning organisation. |
| SUS Grouper Version | SUS_Grouper_Version | The version of the national Payment Grouper used to derive HRGs for the attendance. |
| SUS Code Cleaning Applied | SUS_Code_Cleaning_Applied | Indicates whether national SUS code‑cleaning rules were applied to the record. |
| SUS HRG Code | SUS_HRG_Code | The Healthcare Resource Group assigned to the attendance for costing and payment. |
| SUS Costing Period | SUS_Costing_Period | The costing period used by SUS to calculate the tariff for the attendance. |
| SUS Excluded Flag | SUS_Excluded | Indicates whether the attendance was excluded from SUS processing or payment. |
| SUS Tariff | SUS_Tariff | The national tariff price applied to the attendance based on the HRG. |
| SUS Tariff Description | SUS_Tariff_Description | A description of the tariff applied to the attendance. |
| SUS Market Forces Factor | SUS_MFF | The Market Forces Factor applied to adjust the tariff for local cost variations. |
| **Variable** | **Variable Name** | **Definition** |
| SUS MFF Adjustment | SUS_MFAdjustment | The monetary adjustment applied to the tariff based on the MFF. |
| SUS Final Price | SUS_Final_Price | The final calculated price for the attendance after all SUS adjustments. |
| Age Range Category | Age_Range | The age‑band category assigned to the patient for reporting or costing. |
| Chief Complaint (Approved Code) | Clinical_Chief_Complaint_Code_Approved | The approved national code representing the patient’s chief complaint. |
| Chief Complaint Injury‑Related Flag | Clinical_Chief_Complaint_Injury_Related | Indicates whether the chief complaint is related to an injury. |
| Chief Complaint Extended Code | Clinical_Chief_Complaint_Extended_Code | An extended code providing additional detail about the chief complaint. |
| Acuity (Approved Code) | Clinical_Acuity_Code_Approved | The approved national code representing the patient’s clinical acuity. |
| Injury Activity Status (Approved Code) | Clinical_Injury_Activity_Status_Code_Approved | The approved national code describing the patient’s activity status at the time of injury. |
| Injury Activity Type (Approved Code) | Clinical_Injury_Activity_Type_Code_Approved | The approved national code describing the type of activity the patient was engaged in when injured. |
| Injury Intent (Approved Code) | Clinical_Injury_Intent_Code_Approved | The approved national code describing the intent behind the injury. |
| Injury Place Type (Approved Code) | Clinical_Injury_Place_Type_Approved | The approved national code describing the type of place where the injury occurred. |
| Expected Chief Complaint Recorded | DQ_Chief_Complaint_Expected | Indicates whether a chief complaint entry was expected for this attendance. |
| Chief Complaint Completed | DQ_Chief_Complaint_Completed | Indicates whether a chief complaint was actually recorded. |
| Chief Complaint Valid | DQ_Chief_Complaint_Valid | Indicates whether the recorded chief complaint meets validity rules. |
| Expected Primary Diagnosis Recorded | DQ_Primary_Diagnosis_Expected | Indicates whether a primary diagnosis entry was expected for this attendance. |
| Primary Diagnosis Completed | DQ_Primary_Diagnosis_Completed | Indicates whether a primary diagnosis was recorded. |
| Primary Diagnosis Valid | DQ_Primary_Diagnosis_Valid | Indicates whether the recorded primary diagnosis meets validity rules. |
| Exclusion Reason | Exclusions | Identifies whether the record is excluded from certain analyses and the reason why. |
| Reason for Access | Reason_For_Access | Indicates the reason the patient accessed emergency care (e.g., clinical need, referral). |
| **Variable** | **Variable Name** | **Definition** |
| Pseudonymised NHS Number | Der_Pseudo_NHS_Number | A pseudonymised version of the NHS number generated for linkage without revealing identity. |
| Derived Provider Code | Der_Provider_Code | An organisation identifier for the provider of the Urgent and Emergency Care Service. |
| Derived Provider Site Code | Der_Provider_Site_Code | An organisation identifier for the provider site where the Urgent and Emergency Care Service is located. |
| Derived Commissioner Code | Der_Commissioner_Code | The commissioner code derived from the patient’s postcode or GP practice. |
| Derived Age at Activity Date | Der_Age_At_CDS_Activity_Date | The patient’s age in years calculated from date of birth and activity date. |
| Derived Activity Month | Der_Activity_Month | The month extracted from the activity date for reporting. |
| Derived Financial Year | Der_Financial_Year | The financial year in which the attendance occurred. |
| Number of A&E Diagnoses | Der_Number_AEA_Diagnosis | The count of diagnosis codes recorded for the A&E attendance. |
| Number of EC Diagnoses | Der_Number_EC_Diagnosis | The count of diagnosis codes recorded for the Emergency Care attendance. |
| Number of A&E Investigations | Der_Number_AEA_Investigation | The number of investigations recorded for the A&E attendance. |
| Number of EC Investigations | Der_Number_EC_Investigation | The number of investigations recorded for the Emergency Care attendance. |
| Number of A&E Treatments | Der_Number_AEA_Treatment | The number of treatments recorded for the A&E attendance. |
| Number of EC Treatments | Der_Number_EC_Treatment | The number of treatments recorded for the Emergency Care attendance. |
| All A&E Diagnoses | Der_AEA_Diagnosis_All | A combined field containing all diagnosis codes recorded for the A&E attendance. |
| All EC Diagnoses | Der_EC_Diagnosis_All | A combined field containing all diagnosis codes recorded for the Emergency Care attendance. |
| All A&E Investigations | Der_AEA_Investigation_All | A combined field containing all investigation codes for the A&E attendance. |
| All EC Investigations | Der_EC_Investigation_All | A combined field containing all investigation codes for the Emergency Care attendance. |
| All A&E Treatments | Der_AEA_Treatment_All | A combined field containing all treatment codes for the A&E attendance. |
| All EC Treatments | Der_EC_Treatment_All | A combined field containing all treatment codes for the Emergency Care attendance. |
| Derived Arrival DateTime | Der_EC_Arrival_Date_Time | A combined date‑time field representing the patient’s arrival moment. |
| **Variable** | **Variable Name** | **Definition** |
| Derived Departure DateTime | Der_EC_Departure_Date_Time | A combined date‑time field representing the patient’s departure moment. |
| Derived EC Duration | Der_EC_Duration | The total time spent in the Emergency Department, calculated from arrival and departure. |
| Derived Postcode CCG | Der_Postcode_CCG_Code | The CCG derived from the patient’s postcode. |
| Derived Postcode PCT | Der_Postcode_PCT_Code | The Primary Care Trust derived from the patient’s postcode (legacy field). |
| Derived Postcode Sector | Der_Postcode_Sector | The postcode sector extracted from the patient’s postcode. |
| Derived Local Authority | Der_Postcode_Local_Auth | The local authority derived from the patient’s postcode. |
| Derived District/Unitary Authority | Der_Postcode_Dist_Unitary_Auth | The district or unitary authority derived from the postcode. |
| Derived Electoral Ward | Der_Postcode_Electoral_Ward | The electoral ward derived from the patient’s postcode. |
| Derived LSOA Code | Der_Postcode_LSOA_Code | The Lower Layer Super Output Area derived from the postcode. |
| Derived MSOA Code | Der_Postcode_MSOA_Code | The Middle Layer Super Output Area derived from the postcode. |
| Derived Constituency Code | Der_Postcode_Constituency_Code | The parliamentary constituency derived from the patient’s postcode. |
| Duplicate Record Flag | Der_Dupe_Flag | Indicates whether the record is considered a duplicate. |
| Derived Record Type | Der_Record_Type | A derived classification indicating the type of record. |
| Pseudonymised Pathway ID | Der_Pseudo_Patient_Pathway_ID | A pseudonymised version of the patient pathway identifier. |
| A&E Patient Group | Der_AEA_Patient_Group | A derived grouping of patients based on A&E attendance characteristics. |
| Derived LSOA 2011 | Der_Postcode_LSOA_2011_Code | The 2011 LSOA derived from the patient’s postcode. |
| Derived MSOA 2011 | Der_Postcode_MSOA_2011_Code | The 2011 MSOA derived from the patient’s postcode. |
| Derived LSOA 2021 | Der_Postcode_LSOA_2021_Code | The 2021 LSOA derived from the patient’s postcode. |
| Derived MSOA 2021 | Der_Postcode_MSOA_2021_Code | The 2021 MSOA derived from the patient’s postcode. |
| Clinically Ready Timestamp | Clinically_Ready_To_Proceed_Timestamp | The date‑time when the patient was clinically ready to proceed to the next stage of care. |
| Time to Clinical Readiness | Clinically_Ready_To_Proceed_Time_Since_Arrival | The time between arrival and the patient becoming clinically ready to proceed. |
| Ambulance Care Contact ID | Ambulance_Care_Contact_Identifier | A derived identifier linking the attendance to an ambulance care contact. |
| **Variable** | **Variable Name** | **Definition** |
| Disease Notification Code | Clinical_Disease_Notification_Code | The code representing the type of statutory disease notification. |
| Disease Notification Description | Clinical_Disease_Notification_Description | A description of the statutory disease notification type. |
| Ethnic Category (2021) | Ethnic_Category_2021 | The patient’s ethnicity coded using the 2021 classification. |

**Table 3: Variables used in the CART analysis. Definitions taken from (NHS England, 2025).**

| **Variable** | **Variable Name** | **Definition** |
| --- | --- | --- |
| Age at Arrival | Age_At_Arrival | The age of the patient on date the activity took place. Derived as the number of complete years between the patient’s birth date and the activity date. |
| Sex | Sex | The person phenotypic sex of the patient  (1 = “Male”, 2 = “Female”,  9 = “Indeterminate”, X = “Not Known”). |
| IMD Decile | Index_of_Multiple_Deprivation_Decile | A measure of relative deprivation based on where the patient resides. Areas are ranked based on their overall deprivation score and divided into deciles (1 = “Most deprived areas”, 10 = “Least deprived areas”). |
| Hour | Hour | The hour of the day the patient arrived at the Urgent and Emergency Care Service. Calculated from the Arrival Time. |
| Day of Week | Day of Week | The day of the week the patient arrived at the Urgent and Emergency Care Service. Calculated from the Arrival Date. |
| Emergency Department Type | EC_Department_Type | The type of emergency care department (01 = “Major Emergency Care Department”, 02 = “Mono-specialty Emergency Care Department”,  03 = “Urgent Treatment Centre”,  04 = “NHS Walk-In Centre”). |
| Derived Provider Site Code | Der_Provider_Site_Code | An organisation identifier for the provider site where the Urgent and Emergency Care Service is located. |
| **Variable** | **Variable Name** | **Definition** |
| Distance: LSOA to Provider | Attendance_LSOA_Provider_Distance | The distance between the patient’s LSOA of residence and the provider site. |
| Arrival Mode (SNOMED) | EC_Arrival_Mode_SNOMED_CT | The SNOMED CT code describing the mode of transport the patient used to arrive (e.g., ambulance or walk-in). |
| Attendance Source (SNOMED) | EC_Attendance_Source_SNOMED_CT | The SNOMED CT code describing the source from which the patient was referred or arrived. |
| Chief Complaint (SNOMED) | EC_Chief_Complaint_SNOMED_CT | The SNOMED CT code describing the nature of the patient’s chief complaint when first assessed by a care professional at the Urgent and Emergency Care Service. |
| Acuity (SNOMED) | EC_Acuity_SNOMED_CT | The SNOMED CT code describing the severity of the patient’s condition at the time of initial assessment at the Urgent and Emergency Care Service.  (1 = “Immediate care level emergency care”,  2 = “Very urgent level emergency care”,  3 = “Urgent level emergency care”  4 = “Standard level emergency care”  5 = “Low acuity level emergency care”). |
| Time to Initial Assessment | EC_Initial_Assessment_Time_Since_Arrival | The time between the patient’s arrival and their initial clinical assessment. |
| Number of EC Investigations | Der_Number_EC_Investigation | The number of clinical investigations recorded for the Emergency Care attendance. |
| Number of EC Treatments | Der_Number_EC_Treatment | The number of clinical treatments recorded for the Emergency Care attendance. |
| Number of EC Diagnosis | Der_Number_EC_Diagnosis | The count of diagnosis codes recorded for the Emergency Care attendance. |
| **Variable** | **Variable Name** | **Definition** |
| Total Attendances | Total_attendances | The total number of attendances to an Urgent and Emergency Care Service the patient had between 1^st^ April 2023 – 31^st^ March 2024. Calculated from the number of ECDS records per patient during this period. |
| Derived EC Duration | Der_EC_Duration | The total time spent in the Emergency Department, calculated from arrival and departure. |
| Discharge Destination (SNOMED) | Discharge_Destination_SNOMED_CT | The SNOMED CT code describing the intended destination of the patient following discharge. |
| Target | Target | A numeric representation of the outcome variable (1 = “Unknown ethnicity”, 0 = “Known ethnicity”). |

**Table 4: The percentage of ECDS records by ethnic category.**

| **Ethnic Category** | **ECDS Records** | |
| --- | --- | --- |
|  | **Percentage (%)** | **Count (n)** |
| Asian or Asian British | 7.99% | 1,929,766 |
| Black or Black British | 4.20% | 1,014,489 |
| Mixed | 2.02% | 488,315 |
| Other ethnic groups | 3.82% | 924,369 |
| White | 67.06% | 16,206,553 |
| Not Known | 5.67% | 1,370,057 |
| Not Stated | 7.75% | 1,872,227 |
| Null | 1.50% | 361,378 |
| **Total** | - | **24,167,154** |

**Table 5: The relative importance of each variable in the CART model for each organisation type, which demonstrates both its predictive power and contribution as a surrogate variable (Lewis, 2000). Higher values are considered more important.**

| **Variable** | **Acute Trusts** | **Community Trusts** | **Independent Sector Organisations** |
| --- | --- | --- | --- |
| Age at Arrival | **-** | - | - |
| Sex | - | - | - |
| IMD Decile | 1 | - | 2 |
| Hour | - | - | - |
| Day of Week | - | - | - |
| Emergency Department Type | 1 | - | - |
| Derived Provider Site Code | 94 | 84 | 78 |
| Distance: LSOA to Provider | - | 3 | - |
| Arrival Mode (SNOMED) | - | - | - |
| Attendance Source (SNOMED) | 1 | 5 | - |
| Chief Complaint (SNOMED) | 1 | 3 | 3 |
| Acuity (SNOMED) | - | - | 8 |
| Time to Initial Assessment | - | - | 6 |
| Number of EC Investigations | 3 | - | - |
| Number of EC Treatments | - | 4 | 3 |
| Number of EC Diagnosis | - | - | - |
| Total Attendances | - | - | - |
| Derived EC Duration | - | 2 | - |
| Discharge Destination (SNOMED) | - | - | - |
